# Supplementary material for: Tracking Migraine Symptoms: A Longitudinal Comparison of Smartphone-Based Headache Diaries and Clinical Interviews
Source: Neurol Int. 2025 Feb 24;17(3):33. doi: 10.3390/neurolint17030033 (PMC11944553; doi:10.3390/neurolint17030033)
Supplement: Supplementary file 1 [file neurolint-17-00033-s001.zip › neurolint-3435917-supplementary.pdf]

Table S1: Descriptive statistics of reported free text (extra) symptoms during headache registration.

symptom name	number of headaches	number of patients
Tingling arm and face	13	2
Vivid dreams	9	1
Tinnitus	7	2
Warm head	6	1
Blurred vision	5	1
Night sweats	4	1
Pressure behind the eye	4	1
Painful jaw joint	3	1
Pulsating when getting up	3	1
Aura	3	2
Gnawing pain	2	1
Stabbing pain	2	1
Cold sensation	2	1
Throbbing pain	1	1
Diarrhea	1	1
Very intense pain	1	1
Blushing	1	1

Table S2: number of participants (percentage) with presence of different migraine associated symptoms during the different phases of migraine. Information was obtained during the intake interview.

Symptom	Any Phase	Premonitory Phase	Ictal Phase	Postdromal phase
**ICHD-3**				
Conjunctival injection	4 (14.8%)	2 (7.4%)	3 (11.1%)	2 (7.4%)
Eyelid oedema	4 (14.8%)	4 (14.8%)	3 (11.1%)	1 (3.7%)
Forehead and facial sweating	9 (33.3%)	4 (14.8%)	8 (29.6%)	0 (0.0%)
Lacrimation	6 (22.2%)	2 (7.4%)	4 (14.8%)	0 (0.0%)
Miosis	2 (7.4%)	2 (7.4%)	1 (3.7%)	0 (0.0%)
Motion sensitivity	17 (63.0%)	5 (18.5%)	16 (59.3%)	3 (11.1%)
Nasal congestion	6 (22.2%)	6 (22.2%)	5 (18.5%)	0 (0.0%)
Nausea	23 (85.2%)	11 (40.7%)	22 (81.5%)	5 (18.5%)
Pain aggravation during routine physical activity(*)	/	/	/	/
Phonophobia	22 (81.5%)	8 (29.6%)	20 (74.1%)	5 (18.5%)
Photophobia	21 (77.8%)	7 (25.9%)	21 (77.8%)	4 (14.8%)
Ptosis	5 (18.5%)	1 (3.7%)	4 (14.8%)	0 (0.0%)
Restlessness or agitation	9 (33.3%)	4 (14.8%)	5 (18.5%)	2 (7.4%)
Rhinorrhea	7 (25.9%)	2 (7.4%)	6 (22.2%)	1 (3.7%)
Throbbing headache(**)	17 (65.4%)	/	17 (65.4%)	/
Vomiting	12 (44.4%)	1 (3.7%)	12 (44.4%)	0 (0.0%)
**Non-ICHD-3**				
Anxiety	4 (14.8%)	1 (3.7%)	4 (14.8%)	1 (3.7%)
Constipation	4 (14.8%)	1 (3.7%)	2 (7.4%)	2 (7.4%)
Craving sweet or salty food	13 (48.1%)	8 (29.6%)	3 (11.1%)	5 (18.5%)
Decreased ability to make sentences	15 (55.6%)	9 (33.3%)	14 (51.9%)	6 (22.2%)
Decreased ability to remember	22 (81.5%)	13 (48.1%)	22 (81.5%)	11 (40.7%)
Decreased ability to speak	16 (59.3%)	7 (25.9%)	15 (55.6%)	5 (18.5%)
Decreased interest in daily activities	26 (96.3%)	8 (29.6%)	25 (92.6%)	9 (33.3%)
Depressed mood	8 (29.6%)	4 (14.8%)	6 (22.2%)	4 (14.8%)
Diarrhea	7 (25.9%)	0 (0.0%)	5 (18.5%)	2 (7.4%)
Excessive thirst	8 (29.6%)	2 (7.4%)	6 (22.2%)	3 (11.1%)
External vertigo	11 (40.7%)	4 (14.8%)	7 (25.9%)	1 (3.7%)
Fatigue	27 (100.0%)	15 (55.6%)	21 (77.8%)	20 (74.1%)
Feeling elated or happy	8 (29.6%)	1 (3.7%)	0 (0.0%)	7 (25.9%)
Feeling exhausted	25 (92.6%)	10 (37.0%)	18 (66.7%)	19 (70.4%)
Flushed face	6 (22.2%)	1 (3.7%)	5 (18.5%)	2 (7.4%)

Frequent urination	7 (25.9%)	2 (7.4%)	6 (22.2%)	2 (7.4%)
Hoarseness	5 (18.5%)	1 (3.7%)	4 (14.8%)	0 (0.0%)
Hypersalivation	1 (3.7%)	1 (3.7%)	1 (3.7%)	0 (0.0%)
Hypersensitivity of face skin	7 (25.9%)	1 (3.7%)	7 (25.9%)	0 (0.0%)
Hypersensitivity of neck skin	9 (33.3%)	4 (14.8%)	8 (29.6%)	3 (11.1%)
Hypersensitivity of scalp skin	11 (40.7%)	3 (11.1%)	10 (37.0%)	3 (11.1%)
Impaired concentration	27 (100.0%)	15 (55.6%)	27 (100.0%)	11 (40.7%)
Internal vertigo	13 (48.1%)	3 (11.1%)	12 (44.4%)	2 (7.4%)
Irritability	23 (85.2%)	12 (44.4%)	22 (81.5%)	5 (18.5%)
Lightheadedness	18 (66.7%)	7 (25.9%)	12 (44.4%)	8 (29.6%)
Loss of appetite	17 (63.0%)	5 (18.5%)	16 (59.3%)	6 (22.2%)
Oliguria	3 (11.1%)	0 (0.0%)	3 (11.1%)	0 (0.0%)
Osmophobia	12 (44.4%)	6 (22.2%)	11 (40.7%)	2 (7.4%)
Pale face	17 (63.0%)	7 (25.9%)	16 (59.3%)	3 (11.1%)
Polyuria	5 (18.5%)	2 (7.4%)	3 (11.1%)	2 (7.4%)
Stiffness of neck	24 (88.9%)	16 (59.3%)	19 (70.4%)	10 (37.0%)
Stomachache	7 (25.9%)	2 (7.4%)	5 (18.5%)	2 (7.4%)
Swollen feeling in mouth	1 (3.7%)	0 (0.0%)	1 (3.7%)	0 (0.0%)
Swollen feeling in throat	1 (3.7%)	0 (0.0%)	1 (3.7%)	1 (3.7%)
Urge to move	6 (22.2%)	4 (14.8%)	4 (14.8%)	1 (3.7%)
yawning	18 (66.7%)	13 (48.1%)	12 (44.4%)	4 (14.8%)

(*): Pain aggravation was not asked within the intake questionnaire, but was available during headache event registration

(**): Throbbing headache was not asked within the intake questionnaire, but during patient-physician history taking at the same moment the intake questionnaire was filled in. Missing data was present for one participant.

Table S3: Agreement per symptom, means weighted at the level of participants.
Agreement was defined as the consistency between the symptom's presence during the intake interview and its presence during headache event registration, denoted as "present" (intake) and "present" (headache event), or "not present" (intake) and "not present" (headache event). Values were calculated as the average of the per-participant mean consistency between reported symptom presence during intake (any phase of the migraine) and the recorded headache events.

Symptom	All headache events	Definite Migraine	Probable migraine	Not migraine
**ICHD-3**				
Conjunctival injection	85.3% / 27 users / 505 events	86.6% / 17 users / 74 events	85.9% / 21 users / 155 events	84.9% / 25 users / 276 events
Eyelid oedema	87.7% / 27 users / 505 events	92.0% / 17 users / 74 events	91.8% / 21 users / 155 events	87.2% / 25 users / 276 events
Forehead and facial sweating	69.7% / 27 users / 505 events	65.5% / 17 users / 74 events	68.8% / 21 users / 155 events	65.9% / 25 users / 276 events
Lacrimation	76.2% / 27 users / 505 events	76.3% / 17 users / 74 events	74.6% / 21 users / 155 events	74.4% / 25 users / 276 events
Miosis	92.5% / 27 users / 505 events	93.6% / 17 users / 74 events	90.5% / 21 users / 155 events	92.0% / 25 users / 276 events
Motion sensitivity	50.7% / 27 users / 505 events	60.7% / 17 users / 74 events	49.3% / 21 users / 155 events	45.6% / 25 users / 276 events
Nasal congestion	79.9% / 27 users / 505 events	89.2% / 17 users / 74 events	79.2% / 21 users / 155 events	75.0% / 25 users / 276 events
Nausea	44.4% / 27 users / 505 events	65.9% / 17 users / 74 events	53.6% / 21 users / 155 events	27.8% / 25 users / 276 events
pain aggravation during routine physical activity (*)	/	/	/	/
Phonophobia	40.7% / 27 users / 505 events	56.2% / 17 users / 74 events	55.1% / 21 users / 155 events	31.8% / 25 users / 276 events
Photophobia	50.8% / 27 users / 505 events	63.5% / 17 users / 74 events	63.6% / 21 users / 155 events	41.3% / 25 users / 276 events
Ptosis	87.1% / 27 users / 505 events	88.2% / 17 users / 74 events	86.2% / 21 users / 155 events	85.1% / 25 users / 276 events
Restlessness or agitation	74.2% / 27 users / 505 events	79.8% / 17 users / 74 events	70.6% / 21 users / 155 events	73.7% / 25 users / 276 events
Rhinorrhea	74.0% / 27 users / 505 events	76.6% / 17 users / 74 events	76.5% / 21 users / 155 events	70.6% / 25 users / 276 events
Throbbing headache	45.2% / 26 users / 447 events	54.1% / 16 users / 73 events	45.3% / 20 users / 151 events	38.4% / 24 users / 223 events
Vomiting	58.0% / 27 users / 505 events	49.1% / 17 users / 74 events	52.8% / 21 users / 155 events	56.2% / 25 users / 276 events
**Non-ICHD-3**				
Anxiety	0.0% / 4 users / 20 events	0.0% / 1 users / 1 events	0.0% / 3 users / 7 events	0.0% / 3 users / 12 events
Constipation	2.0% / 4 users / 40 events	10.0% / 2 users / 9 events	2.2% / 3 users / 19 events	0.0% / 4 users / 12 events
Craving sweet or salty food	13.0% / 13 users / 261 events	26.7% / 10 users / 58 events	18.7% / 12 users / 105 events	6.7% / 12 users / 98 events
Decreased ability to make sentences	16.2% / 15 users / 319 events	16.8% / 12 users / 60 events	8.5% / 13 users / 109 events	10.2% / 14 users / 150 events
Decreased ability to remember	11.7% / 22 users / 390 events	20.8% / 13 users / 66 events	16.5% / 17 users / 128 events	2.7% / 20 users / 196 events

Decreased ability to speak	10.7% / 16 users / 378 events	6.1% / 13 users / 52 events	5.3% / 14 users / 119 events	9.6% / 16 users / 207 events
Decreased interest in daily activities	20.3% / 26 users / 490 events	41.8% / 16 users / 62 events	21.0% / 20 users / 152 events	18.1% / 25 users / 276 events
Depressed mood	20.0% / 8 users / 122 events	26.9% / 5 users / 24 events	16.5% / 5 users / 52 events	22.8% / 8 users / 46 events
Diarrhea	4.9% / 7 users / 143 events	3.0% / 3 users / 17 events	7.4% / 5 users / 40 events	1.6% / 7 users / 86 events
Excessive thirst	19.6% / 8 users / 200 events	20.2% / 6 users / 43 events	21.8% / 8 users / 75 events	23.0% / 8 users / 82 events
External vertigo	7.4% / 11 users / 239 events	12.1% / 6 users / 32 events	9.1% / 9 users / 89 events	7.3% / 11 users / 118 events
Fatigue	53.1% / 27 users / 505 events	71.5% / 17 users / 74 events	60.0% / 21 users / 155 events	48.3% / 25 users / 276 events
Feeling elated or happy	0.0% / 7 users / 130 events	0.0% / 4 users / 24 events	0.0% / 6 users / 46 events	0.0% / 7 users / 60 events
Feeling exhausted	38.3% / 25 users / 494 events	65.1% / 16 users / 70 events	40.8% / 19 users / 152 events	28.3% / 23 users / 272 events
Flushed face	5.4% / 6 users / 141 events	11.4% / 4 users / 24 events	5.3% / 6 users / 52 events	3.4% / 6 users / 65 events
Frequent urination	14.8% / 7 users / 194 events	75.8% / 3 users / 13 events	19.0% / 6 users / 46 events	14.2% / 6 users / 135 events
Hoarseness	1.9% / 5 users / 153 events	4.5% / 2 users / 13 events	1.2% / 5 users / 54 events	1.8% / 5 users / 86 events
Hypersalivation	0.0% / 1 users / 40 events	0.0% / 1 users / 2 events	0.0% / 1 users / 16 events	0.0% / 1 users / 22 events
Hypersensitivity of face skin	14.5% / 7 users / 173 events	23.6% / 3 users / 12 events	15.4% / 5 users / 40 events	12.1% / 7 users / 121 events
Hypersensitivity of neck skin	13.4% / 9 users / 156 events	28.0% / 5 users / 26 events	24.6% / 7 users / 55 events	10.6% / 9 users / 75 events
Hypersensitivity of scalp skin	19.6% / 11 users / 260 events	31.8% / 8 users / 49 events	28.1% / 10 users / 82 events	8.2% / 10 users / 129 events
Impaired concentration	34.0% / 27 users / 505 events	48.1% / 17 users / 74 events	42.3% / 21 users / 155 events	26.1% / 25 users / 276 events
Internal vertigo	15.0% / 13 users / 223 events	20.9% / 8 users / 45 events	24.5% / 11 users / 93 events	7.3% / 12 users / 85 events
Irritability	16.5% / 23 users / 440 events	23.8% / 15 users / 62 events	22.0% / 18 users / 128 events	13.0% / 21 users / 250 events
Lightheadedness	31.4% / 18 users / 343 events	31.9% / 11 users / 54 events	26.7% / 14 users / 118 events	30.3% / 16 users / 171 events
Loss of appetite	18.3% / 17 users / 365 events	40.6% / 13 users / 69 events	23.0% / 14 users / 133 events	10.6% / 16 users / 163 events
Oliguria	4.4% / 3 users / 65 events	0.0% / 1 users / 1 events	0.0% / 3 users / 15 events	5.5% / 3 users / 49 events
Pale face	22.1% / 17 users / 292 events	22.0% / 10 users / 44 events	11.6% / 11 users / 101 events	22.0% / 17 users / 147 events
Polyuria	8.6% / 5 users / 82 events	50.0% / 2 users / 5 events	12.5% / 4 users / 9 events	10.4% / 4 users / 68 events
Stiffness of neck	36.8% / 24 users / 497 events	44.0% / 16 users / 72 events	38.8% / 20 users / 154 events	28.6% / 22 users / 271 events

Stomach ache	27.8% / 7 users / 167 events	50.6% / 6 users / 21 events	29.0% / 7 users / 46 events	20.4% / 7 users / 100 events
Swollen feeling in mouth	4.0% / 1 users / 25 events	0.0% / 1 users / 5 events	6.7% / 1 users / 15 events	0.0% / 1 users / 5 events
Swollen feeling in throat	0.0% / 1 users / 2 events	0.0% / 1 users / 1 events	/	0.0% / 1 users / 1 events
Urge to move	2.6% / 6 users / 140 events	0.0% / 4 users / 17 events	8.3% / 6 users / 47 events	2.2% / 6 users / 76 events
Yawning	25.9% / 18 users / 389 events	27.5% / 11 users / 45 events	21.2% / 15 users / 126 events	26.4% / 17 users / 218 events

(*): Pain aggravation was not asked within the intake questionnaire, but was available during headache event registration

Table S4: Mean reporting (once) ratio of ICHD-3 symptoms; subgroup: "definite migraine".

Symptom	Intake value	Number of participants / Number of headache registrations		Mean symptom reporting ratio (%) (SD)	Participant ratio who reported the symptom at least once
Conjunctival injection	Present	2	9	0.0% (0.0)	0.0% (0/2)
	Not present	15	65	1.8% (7.0)	6.7% (1/15)
Eyelid oedema	Present	2	19	31.8% (45.0)	50.0% (1/2)
	Not present	15	55	0.0% (0.0)	0.0% (0/15)
Forehead and facial sweating	Present	7	33	16.3% (17.6)	57.1% (4/7)
	Not present	10	41	0.0% (0.0)	0.0% (0/10)
Lacrimation	Present	4	13	12.5% (25.0)	25.0% (1/4)
	Not present	13	61	4.0% (8.3)	23.1% (3/13)
Miosis	Present	1	3	0.0% (nan)	0.0% (0/1)
	Not present	16	71	0.6% (2.3)	6.2% (1/16)
Motion sensitivity	Present	12	61	48.4% (37.1)	75.0% (9/12)
	Not present	5	13	10.0% (22.4)	20.0% (1/5)
Nasal congestion	Present	3	21	50.0% (50.0)	66.7% (2/3)
	Not present	14	53	2.4% (6.3)	14.3% (2/14)
Nausea	Present	15	72	74.7% (37.1)	86.7% (13/15)
	Not present	2	2	100.0% (0.0)	100.0% (2/2)
Phonophobia	Present	15	71	50.4% (44.3)	66.7% (10/15)
	Not present	2	3	0.0% (0.0)	0.0% (0/2)
Photophobia	Present	15	71	62.0% (45.4)	73.3% (11/15)
	Not present	2	3	25.0% (35.4)	50.0% (1/2)
Ptosis	Present	2	12	0.0% (0.0)	0.0% (0/2)
	Not present	15	62	0.0% (0.0)	0.0% (0/15)
Restlessness or agitation	Present	4	17	34.4% (23.7)	75.0% (3/4)
	Not present	13	57	6.2% (11.8)	23.1% (3/13)
Rhinorrhea	Present	4	23	13.9% (17.9)	50.0% (2/4)
	Not present	13	51	4.2% (8.6)	23.1% (3/13)
Throbbing headache	Present	12	56	47.1% (44.7)	66.7% (8/12)
	Not present	4	17	25.0% (28.9)	50.0% (2/4)
Vomiting	Present	9	36	26.0% (42.6)	44.4% (4/9)
	Not present	8	38	25.0% (46.3)	25.0% (2/8)

Table S5: Mean reporting (once) ratio of ICHD-3 symptoms, Subgroup: “definite migraine” and “probable migraine”.					
**Symptom**	**Intake value**	**Number of participants / Number of headache registrations**		**Mean symptom reporting ratio (%) (SD)**	**Participant ratio who reported the symptom at least once**
Conjunctival injection	Present	3	39	4.2% (7.2)	33.3% (1/3)
	Not present	19	190	0.9% (3.2)	10.5% (2/19)
Eyelid oedema	Present	2	52	28.9% (34.9)	100.0% (2/2)
	Not present	20	177	1.2% (5.0)	10.0% (2/20)
Forehead and facial sweating	Present	8	114	13.3% (12.7)	62.5% (5/8)
	Not present	14	115	0.0% (0.0)	0.0% (0/14)
Lacrimation	Present	6	50	16.7% (27.9)	33.3% (2/6)
	Not present	16	179	5.5% (12.8)	31.2% (5/16)
Miosis	Present	2	14	0.0% (0.0)	0.0% (0/2)
	Not present	20	215	0.1% (0.6)	5.0% (1/20)
Motion sensitivity	Present	15	173	35.2% (33.4)	73.3% (11/15)
	Not present	7	56	9.2% (13.2)	42.9% (3/7)
Nasal congestion	Present	5	62	53.9% (41.7)	100.0% (5/5)
	Not present	17	167	9.3% (16.8)	35.3% (6/17)
Nausea	Present	20	216	56.2% (36.5)	80.0% (16/20)
	Not present	2	13	36.2% (33.6)	100.0% (2/2)
Phonophobia	Present	19	212	44.7% (37.5)	78.9% (15/19)
	Not present	3	17	0.0% (0.0)	0.0% (0/3)
Photophobia	Present	18	211	55.6% (37.7)	83.3% (15/18)
	Not present	4	18	3.6% (7.1)	25.0% (1/4)
Ptosis	Present	4	45	28.7% (22.2)	100.0% (4/4)
	Not present	18	184	0.5% (2.0)	5.6% (1/18)
Restlessness or agitation	Present	7	67	22.3% (19.0)	71.4% (5/7)
	Not present	15	162	3.8% (7.7)	26.7% (4/15)
Rhinorrhea	Present	6	73	17.0% (15.1)	66.7% (4/6)
	Not present	16	156	4.7% (7.1)	37.5% (6/16)
Throbbing headache	Present	14	156	31.9% (29.8)	85.7% (12/14)
	Not present	7	68	7.1% (12.3)	42.9% (3/7)
Vomiting	Present	11	113	13.9% (30.2)	36.4% (4/11)
	Not present	11	116	4.8% (12.3)	18.2% (2/11)

In examining the "Mean symptom reporting ratio" column from the referenced table, it is evident that nausea, photophobia, and phonophobia are the most consistently reported symptoms, exhibiting a high incidence rate. This observation is in line with the findings of Lieba-Samal et al. (22). Additionally, our data indicate that nasal congestion is another stable symptom. Notably, nausea was reported even by patients who initially did not identify this symptom during intake (i.e., those with an “intake value” of False), suggesting a potential underreporting of this symptom. On the other hand, conjunctival injection and miosis emerge as notably rare symptoms, with low incidence rates, even for patients who indicated symptom occurrence during the intake questionnaire
